# EEG-Based Supported Diagnosis of ADHD Using Subject-Specific HMMs and Stationary RKHS Embeddings

**DOI:** 10.3390/s26154773

**Published:** 2026-07-27

**Authors:** Leonardo Lopez-Ortiz, Cristhian K. Valencia-Marin, Julián Gil-González, Paula M. Herrera-Gómez, David Cárdenas-Peña

**Affiliations:** 1 Automatics Research Group, Universidad Tecnológica de Pereira (UTP), Pereira 660003, Colombia; ckvalencia@utp.edu.co (C.K.V.-M.); jugil@utp.edu.co (J.G.-G.); 2 Psiquiatría Neurociencias y Comunidad, Universidad Tecnológica de Pereira (UTP), Pereira 660003, Colombia; p.herrera@utp.edu.co

**Keywords:** EEG, ADHD, hidden Markov model, Gaussian mixture model, RKHS embedding, kernel classification

## Abstract

Electroencephalography (EEG) provides a non-invasive means of supporting Attention-Deficit/Hyperactivity Disorder (ADHD) assessment. Nonetheless, existing pipelines often rely on handcrafted descriptors, segment-wise decisions, or deep architectures with limited subject-level generalization. This work introduces Hidden Markov Model-Induced Stationary RKHS Distance Learning (HIS), a probabilistic framework that represents each subject by a Hidden Markov Model with Gaussian-mixture emissions trained directly from frontal EEG recordings. Rather than vectorizing model parameters, each HMM is mapped to its induced stationary observation distribution and embedded into a Reproducing Kernel Hilbert Space (RKHS), where pairwise subject similarities are computed through a closed-form Hilbert embedding distance. These similarities are subsequently exploited by precomputed-kernel classifiers for subject-level prediction. The proposed method was evaluated against the Probability Product Kernel baseline using both a controlled synthetic EEG benchmark and a public pediatric ADHD dataset under progressively more rigorous validation protocols, culminating in repeated nested cross-validation with bootstrap confidence intervals and permutation testing. On the synthetic benchmark, HIS achieved 95.0% held-out accuracy and consistently outperformed the baseline across classifiers. On a real EEG dataset with 121 subjects, the primary evaluation protocol yielded a balanced accuracy of 73.5% (95% CI: 69.8–77.0%), an AUC of 79.6%, and an MCC of 0.483 (permutation *p* < 0.001) using an SVM with compact subject-specific HMMs. Complementary hyperparameter analyses and t-SNE visualizations demonstrated that HIS induces more stable and discriminative subject representations than the baseline. These results establish stationary RKHS embeddings of subject-specific HMMs as a leakage-aware framework for EEG-based ADHD decision support and underscore the critical influence of statistically rigorous evaluation protocols on reported classification performance.

## 1. Introduction

Attention-Deficit/Hyperactivity Disorder (ADHD) represents one of the most prevalent neurodevelopmental conditions affecting children and adolescents, with substantial consequences for learning, executive functioning, and socio-emotional development [1]. Impulsivity, a central diagnostic dimension, has been linked to academic difficulties, impaired social interaction, and long-term behavioral risks [2]. Recent findings in Latin American pediatric populations highlight additional deficits in metalinguistic skills [3] and working-memory-related social behavior [4]. Electroencephalography (EEG), owing to its high temporal resolution and non-invasive nature, has become a widely adopted tool for studying attentional and executive function abnormalities in ADHD [5]. Traditional EEG analyses rely heavily on handcrafted features such as bandpower ratios, the theta–beta ratio (TBR), entropy-based descriptors, and other statistical or nonlinear measures [6,7,8]. Although these feature engineering approaches can capture relevant aspects of EEG activity, they remain strongly dependent on manual preprocessing decisions and may overlook latent temporal–spectral structure present in the raw signals. Recent methodological reviews emphasize the importance of time-series modeling frameworks capable of representing this rich structure directly from data [9].

In parallel with these handcrafted approaches, deep learning has yielded major advances in EEG-based ADHD analysis. Graph Convolutional Networks with attention mechanisms have revealed atypical functional connectivity patterns [10], while graph-theoretic analyses of EEG connectomes using minimum spanning tree representations have also demonstrated discriminative connectivity alterations between ADHD and neurotypical children [11]. Attention auto-encoding and transformer-based architectures have further achieved high classification performance and identified potential neurobiological markers [12,13]. Numerous architectures reflect the increasing sophistication of deep learning and hybrid EEG systems, including ADHDCD-NET [14], explainable CNN pipelines [15], impulsivity classifiers using power features [16], and channel-selection or regional-contribution models [17,18]. Additional work incorporates fNIRS multimodal learning [19] and underscores the sensitivity of deep models to preprocessing and segmentation choices [20]. More recent attention-driven architectures [21] aim to balance accuracy with interpretability. Despite these advances, deep models often behave as black boxes: their internal representations can be unstable across subjects, preprocessing strategies, and data folds, limiting their clinical explainability and hindering biomarker discovery.

Probabilistic generative models, particularly Hidden Markov Models (HMMs), offer an alternative perspective compared to both handcrafted and deep learning approaches. Unlike feature-based methods or deep networks, HMMs provide a structured representation of EEG dynamics by directly modeling EEG as a sequence of transitions between hidden neural states. These states capture underlying rhythms, activation patterns, and cognitive regimes without relying on manually engineered descriptors or heavily parameterized neural architectures. HMMs have been successfully applied in machinery monitoring, biomedical event detection, interpretable motion analysis, and Wasserstein-based registration. Recent developments have proposed advanced HMM distance measures, including state-aligned metrics and Hilbert-embedding methods for time-series classification. In EEG analysis, interpretable kernel-based HMM approaches have provided an alternative to purely feature-engineered or deep architectures [22]. The latent-state structure of HMMs provides explicit probabilistic quantities, including transition probabilities, state occupancies, and Gaussian emission statistics, that can be analyzed after model fitting. These quantities offer a structural transparency that may support future neurophysiological interpretation.

The proposed approach addresses three limitations. First, handcrafted EEG features may overlook latent temporal structure and remain dependent on manual design choices. Second, existing integrations of subject-specific HMM–GMM representations with Reproducing Kernel Hilbert Space (RKHS) embeddings rely on approximate similarity measures, motivating a theoretically grounded distance between stationary observation distributions induced by the underlying generative models. Finally, many reported ADHD classification results have been evaluated under validation protocols that may overestimate generalization performance due to segment-level partitioning or non-nested model selection. The present work therefore combines a stationary RKHS representation of subject-specific HMMs with a subject-level repeated nested cross-validation protocol to provide a more robust assessment of generalization while preserving the probabilistic structure learned from variable-length EEG recordings.

This study builds upon recent work on Hilbert-embedded HMM similarity metrics [23,24,25] by integrating them into a unified probabilistic–kernel framework tailored for multichannel EEG. Our aim is to advance this line of research by adapting existing mathematical developments to the specific characteristics of pediatric ADHD. Our contribution is a principled adaptation of these distance measures to subject-specific HMM–GMM models trained on frontal EEG from children with ADHD and typically developing controls. In parallel, this work develops a practical matrix-form implementation that enables efficient computation of pairwise distances across participants. Drawing on insights from EEG preprocessing and feature selection methodologies [17,18], the designed pipeline emphasizes the relevance of frontal channels while avoiding handcrafted feature engineering.

The resulting framework combines generative modeling, Hilbert-space embeddings, and precomputed-kernel classification (KNN and SVM) into a reproducible workflow suitable for clinical EEG data. The approach is evaluated both on synthetic EEG signals constructed to emulate ADHD-related spectral characteristics and on real frontal-channel EEG recordings from children aged 7–12 years. Across both datasets, the adapted RKHS-based distance yields structured similarity matrices and robust classification performance metrics. In the real-data analysis, the proposed metric demonstrates statistically robust generalization on held-out subjects and provides advantages relative to feature-based and PPK baselines, suggesting that Hilbert-embedded HMM distances constitute a promising direction for EEG-based ADHD characterization.

## 2. Materials and Methods

The proposed methodology in this study, hereafter referred to as HMM-Induced Stationary RKHS Distance Learning (HIS), comprises four main stages: (i) EEG preprocessing and channel selection, (ii) subject-specific HMM–GMM model estimation, (iii) RKHS-based pairwise distance computation, and (iv) classification using precomputed kernel methods.

### 2.1. Synthetic EEG Benchmark Signals

Prior to evaluating the RKHS-based distance metric on real ADHD EEG recordings, a controlled synthetic experiment is conducted to assess the behavior of the Hilbert-embedded HMM distance under fully specified spectral conditions. Simulated EEG signals provide ground-truth neural activity and allow systematic exploration of how spectral content, noise, and artifacts affect algorithm performance [26]. In particular, the synthetic benchmark serves as a sanity check and methodological stress test for the proposed HIS distance under controlled spectral differences and artifact perturbations. Thus, the data-generating process below yields a deliberately simplified classification problem that illustrates the discriminative capacity of the proposed approach before it is applied to real pediatric EEG data with clinical or neurophysiological validity.

Let $S^{\mathrm{synth}}=\left\{s_{1}^{\mathrm{synth}},\ldots ,s_{N}^{\mathrm{synth}}\right\}$ denote the set of N=100 synthetic subjects, with binary labels $y_{s}^{\mathrm{synth}}\in \left\{-1,1\right\}$ indicating ADHD-diagnosed ($y_{s}^{\mathrm{synth}}=1$) or healthy-control ($y_{s}^{\mathrm{synth}}=-1$) individuals. The EEG signals are simulated as a balanced dataset of univariate time series $x_{s}^{\mathrm{synth}}\in R^{1\times T}$ lasting T=3840 samples, corresponding to a 30-s recording segment at a sampling frequency $f_{s}=128$ Hz. Each synthetic signal is designed as a weighted sum of sinusoidal components in the canonical EEG frequency bands plus additive Gaussian noise [27]:

(1) $$x_{s}^{\mathrm{synth}}\left(t\right)=\sum_{b\in B}A_{sb}\sin\left(2\pi f_{sb}t+\phi_{sb}\right)+\epsilon_{s}\left(t\right),$$

where B={δ,θ,α,β} denotes the set of EEG bands or brain rhythms defined as delta [0.5,4] Hz, theta [4,7] Hz, alpha [8,13] Hz, and beta [14,30] Hz, respectively; $A_{sb}\in R^{+}$ is the amplitude weight for band *b*; $f_{sb}\in R^{+}$ is a randomly perturbed center frequency within band *b*; $\phi_{sb}∼U\left(0,2\pi \right)$ is a phase randomly sampled from a uniform distribution; and $\epsilon_{s}\left(t\right)∼N\left(0,\sigma_{s}^{2}\right)$ introduces additive zero-mean Gaussian noise with subject-dependent variance $\sigma_{s}^{2}\in R^{+}$. Since the EEG literature reports increased slow-wave (delta/theta) and decreased fast-wave (alpha/beta) activity in ADHD relative to control populations [28,29], class-specific parameters control band-amplitudes in the simulated categories. For control signals ($y_{s}^{\mathrm{synth}}=0$), slow-rhythm amplitudes are suppressed and fast-rhythm amplitudes highlighted:

$$A_{s\delta },A_{s\theta }∼U\left(0.3,0.5\right),\;A_{s\alpha },A_{s\beta }∼U\left(0.8,1.2\right),\;\sigma_{s}∼U\left(0.2,0.4\right).$$

In turn, the pattern is reversed for ADHD signals ($y_{s}^{\mathrm{synth}}=1$):

$$A_{s\delta },A_{s\theta }∼U\left(0.8,1.2\right),\;A_{s\alpha },A_{s,\beta }∼U\left(0.3,0.5\right),\;\sigma_{s}∼U\left(0.4,0.6\right).$$

**Figure 1:**
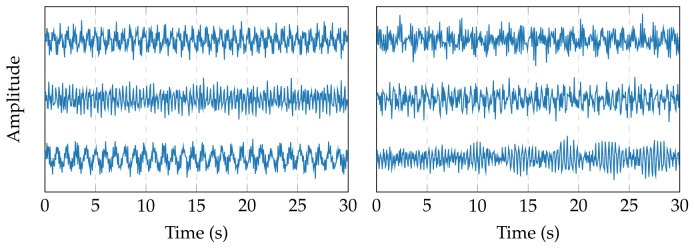
Synthetic EEG samples for three ADHD (**left**) and three Control (**right**) recordings. Traces are vertically offset for visualization.

To emulate occasional non-physiological disturbances, the generator additionally injects brief high-amplitude spikes into the synthetic signals, following prior literature on artifact characteristics in pediatric populations [30]. At each time instant, there is a probability $p_{\mathrm{spike}}=0.05$ of a spike onset of amplitude $A_{\mathrm{spike}}∼U\left(3,5\right)$ (in units of the signal’s standard deviation) lasting 3–5 samples. Finally, a standard scaler normalizes each time series, resulting in centered, variance-constrained signals as exemplified by Figure 1 for each class.

Note that the resulting class-conditional distributions induce differences in the Gaussian-mixture emission structure, allowing assessment of the quality of probability-based distance models in a controlled scenario with spectral structure and artifact perturbations. In particular, the designed spectral difference between classes controls the separability in the dataset $D^{\mathrm{synth}}=\left\{\left(x_{s}^{\mathrm{synth}},y_{s}^{\mathrm{synth}}\right):s\in S^{\mathrm{synth}}\right\}$, allowing to verify that the proposed HIS distance behaves as expected under a known, controlled spectral structure with artifact perturbations. Therefore, the performance on this benchmark, rather than tuned to favor any particular classifier or distance measure, yields a necessary but not sufficient condition for real-data validity.

### 2.2. Real ADHD EEG Dataset

The real dataset for this study, publicly available on IEEE DataPort, contains EEG recordings from 61 children with ADHD and 60 age-matched controls (boys and girls, ages 7–12) [31]. The participants in the ADHD group were diagnosed by an experienced psychiatrist according to DSM-IV criteria, and all had received methylphenidate (Ritalin) for up to six months prior to data acquisition. The control participants had no history of psychiatric disorders, epilepsy, or engagement in high-risk behaviors. This dataset was selected for its standardized EEGs from a well-defined ADHD cohort and matched controls.

**Figure 2:**
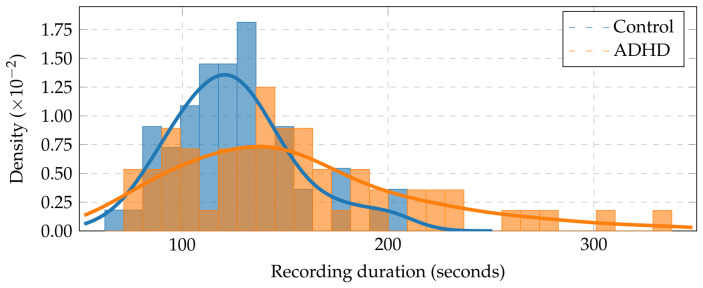
Distribution of recording durations for Control and ADHD groups.

Given that deficits in visual attention are commonly reported in children with ADHD, the recording protocol was designed around a visual attention task. During the task, children were presented with pictures of cartoon characters and were asked to count the number of characters in each image. The number of characters ranged randomly from 5 to 16, and each image was displayed at a size large enough to ensure visibility and accurate counting. To maintain continuous cognitive engagement, each stimulus was presented immediately after the child’s response without interruption. Consequently, the total duration of EEG recording varied across participants, ranging from approximately 50 to 285 s, depending on each child’s response speed. Figure 2 illustrates the recording duration for ADHD and control participants. It is noteworthy that recording durations show distinct distributions across groups. Control recordings are around 100–150 s long, with a relatively narrow distribution, whereas ADHD recordings exhibit a broader, right-skewed distribution extending to substantially longer durations. This indicates greater variability in recording length for the ADHD group. The partial overlap between the two KDE curves suggests that both groups share a common duration range, but the heavier right tail in the ADHD group highlights the need for models that handle unequal sequence lengths.

**Figure 3:**
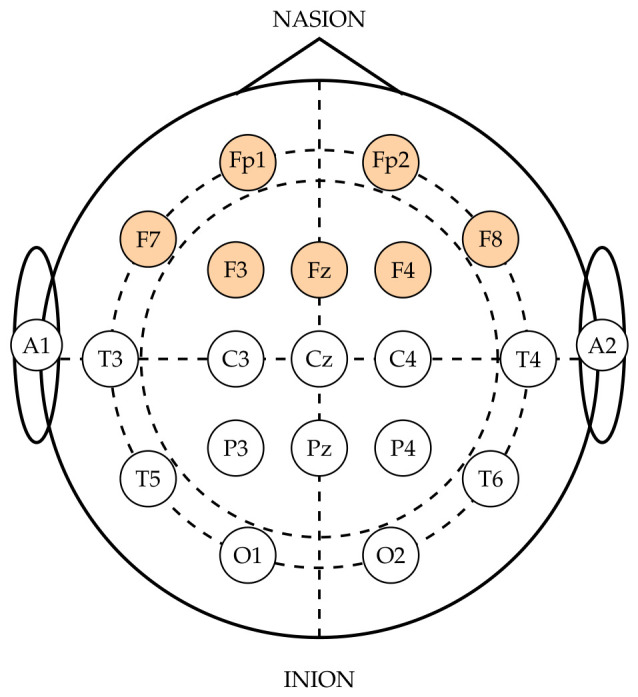
The 10–20 international EEG electrode placement system. Frontal channels used in this study (Fp1, Fp2, F7, F8, F3, Fz, F4) are highlighted in orange.

During the visual attention task, EEG signals were recorded following the international 10–20 system using 19 scalp electrodes (see Figure 3) at a sampling frequency of $f_{s}=128$ Hz. The A1 and A2 electrodes, placed on the earlobes, served as reference channels. For each subject s∈S, the raw EEG recording is represented as a matrix $X\in R^{C\times T_{s}}$, where C=19 denotes the number of recording channels and $T_{s}$ represents the number of time samples for subject *s*. Due to the task-dependent nature of the recording protocol, the duration varied across participants, with $T_{s}\in \left[6400,36480\right]$ samples.

Following the above description, the dataset is mathematically structured as a set of subjects $S=\{s_{1},s_{2},\ldots ,s_{121}\}$, each of them holding a binary label $y_{s}\in \left\{-1,1\right\}$, such that $y_{s}=1$ indicates that subject *s* belongs to the ADHD group, and $y_{s}=-1$ corresponds to a control subject.

### 2.3. Preprocessing

The preprocessing stage enhances signal quality so that the subsequent probabilistic modeling can capture meaningful temporal and spectral information. Prior work on the same ADHD dataset shows the relevance of frontal regions and canonical EEG bands for distinguishing ADHD from controls [32], and frontal channels are particularly informative for attention-related processes and executive function. The analysis is therefore restricted to the $C^{*}=7$ frontal and prefrontal electrodes of the original 19-channel montage, $C^{*}=\left\{\mathrm{Fp}1,\;F7,\;F3,\;\mathrm{Fz},\;F4,\;F8,\mathrm{Fp}2\right\}$ [17,18]. A fourth-order Butterworth bandpass filter (0.5–30 Hz) retains the delta (0.5–4 Hz), theta (4–8 Hz), alpha (8–13 Hz), and beta (13–30 Hz) bands while removing slow drifts and high-frequency muscle noise [33]; a fourth-order 50 Hz notch filter suppresses power-line interference.

Because the montage includes the prefrontal electrodes Fp1 and Fp2, which are highly sensitive to eye blinks and ocular movements [34], and because pediatric artifacts may differ between groups, an explicit quality-control screen was applied after filtering. An amplitude inter-quartile-range (IQR) screen clips samples beyond $Q_{1}-3.0\;\mathrm{IQR}$ or $Q_{3}+3.0\;\mathrm{IQR}$, and a channel-variance screen flags channels with |z|>3.5. The screen clipped only about 1.4% of samples per subject on average (ranging from 0.020 to 4.766) with 40/121 subjects showing a flagged channel. It is worth mentioning that the clipped fraction is similar between groups (Control 1.5±1.1%; ADHD 1.3±0.8%; Welch’s t=1.1, p=0.2), arguing against a group-dependent artifact load that the classifier could exploit. Despite detecting anomalous amplitude and variance and finding no difference between groups, this quality control does not replace ocular- or movement-artifact correction. Since the dataset lacks EOG channels, manual annotations, or impedance logs to calibrate such a correction, deeper separation of neural from artifact-related patterns cannot be achieved. Lastly, channel-wise standard scaling enforces a zero mean and unit variance to stabilize HMM training. The preprocessed dataset is then represented as $D^{\mathrm{real}}=\left\{\left(X_{s}^{\mathrm{real}}\in R^{C^{*}\times T_{s}},y_{s}\right):s\in S\right\}$.

### 2.4. Hidden Markov Model-Based Time-Series Representation

To characterize the temporal structure of the EEG recordings, each subject was represented by its own Hidden Markov Model (HMM) with Gaussian mixture emissions. This modeling choice provides a probabilistic description of the latent temporal regimes underlying the multichannel EEG signals, while allowing each regime to account for heterogeneous spectral–spatial patterns through a flexible mixture distribution. For the subject *s*, the preprocessed EEG signal is assumed to follow an HMM distribution

(2) $$x_{s}\left(t\right)|\theta_{s}∼\mathrm{HMM}\left(\pi_{s},A_{s},{\left\{b_{s,i}\left(\cdot \right)\right\}}_{i=1}^{K}\right),$$

being $x_{s}\left(t\right)\in R^{C^{*}}$ the *t*-th time-instant within the subject recording $X_{s}$. The set $\theta_{s}=\left(\pi_{s},A_{s},\phi_{s}\right)$ parameterizes the subject’s HMM with $\pi_{s}=\left[\pi_{s1},\pi_{s2},\ldots ,\pi_{sK}\right]$ as the initial state distribution, accomplishing $\pi_{si}=P\left(q_{1}=i\right)$ and $\sum_{i=1}^{K}\pi_{si}=1$; $A_{s}\in {\left[0,1\right]}^{K\times K}$ denoting the state transition matrix, whose entries satisfy ${\left[A_{s}\right]}_{ij}=P\left(q_{t+1}=j|q_{t}=i\right)$ and $\sum_{j=1}^{K}{\left[A_{s}\right]}_{ij}=1$ for every state *i*; $\phi_{s}={\left\{\phi_{s,i}\right\}}_{i=1}^{K}$ holding the emission parameters. Here, *K* corresponds to the number of hidden states and $q_{t}\in \left\{1,\ldots ,K\right\}$ denotes the latent state at time *t*. Each hidden state is endowed with a Gaussian Mixture Model (GMM) emission density composed of *M* components.

(3) $$p\left(x_{st}|q_{t}=i,\phi_{si}\right)=\sum_{m=1}^{M}w_{sim}\;N\left(x_{st}|\mu_{sim},\Sigma_{sim}\right),$$

where $w_{sim}\in {\left[0,1\right]}^{+}$ is a non-negative mixture weight satisfying $\sum_{m=1}^{M}w_{sim}=1$, $\mu_{sim}\in R^{C^{*}}$ and $\Sigma_{sim}\in R^{C^{*}\times C^{*}}$ correspond to the mean vector and the covariance matrix for the *m*-th Gaussian component N(·|·,·) associated with state *i*, respectively. Hence, the emission parameter set becomes $\phi_{s}={\left\{w_{sim},\mu_{sim},\Sigma_{sim}\right\}}_{i,m=1}^{K,M}$. This formulation enables each latent state to represent a distribution over EEG configurations rather than a single prototypical pattern, thereby improving the capacity of the model to capture nonstationary and heterogeneous neural dynamics across time.

For each subject, the parameters of the HMM are estimated by maximizing the log-likelihood of the EEG recording X using the Baum-Welch (BW) algorithm, which is based on the Expectation-Maximization procedure for HMMs [35]. During training, the EEG recording from each subject is temporally split into a leading 70% training segment and a trailing 30% validation segment. The training segment is used exclusively to fit the subject-specific HMM–GMM parameters according to the BW algorithm. The validation segment, held out from HMM parameter updates, only monitors the log-likelihood to verify that the fitted model generalizes within-subject before being downstreamed to the RHKS representation. In particular, the validation segment introduces a training convergence criterion: the relative improvement in the validation-segment log-likelihood between consecutive EM iterations falls below a fixed tolerance. Then, this 70/30 split operates within each subject’s recording and is therefore independent of the subject-level train/test partitions used for outer-loop model evaluation. Two additional convergence criteria are also considered: First, HMM training also stops if the stationary distribution is reached, i.e., the current transition matrix changes by less than a fixed tolerance between consecutive iterations, indicating that the latent-state dynamics have stabilized. Second, a default of 100 EM iterations is allowed; if this limit is reached without satisfying either criterion above, training also stops.

At convergence, the learned subject-specific representation is denoted

(4) $$\theta_{s}^{*}=\left(\pi_{s}^{*},A_{s}^{*},\phi_{s}^{*}\right)=\arg\max_{\theta_{s}}\log P\left(X_{s}|\theta_{s}\right),$$

and the complete HMM-based representation of the dataset becomes:

(5) $$D^{\mathrm{HMM}}=\left\{\left(\theta_{s}^{*},y_{s}\right):s\in S\right\},$$

The trained subject-wise HMM parameters are treated as structured probabilistic objects rather than flattened, fixed-dimensional feature vectors, thereby preserving the generative and temporal nature of the subject-specific models.

### 2.5. RKHS-Based Distance Between Hidden Markov Models

To compare the subjects in Equation (5), the proposed HIS adopts a Hilbert-space embedding framework by representing each HMM through its induced stationary observation distribution, followed by the embedding of such distribution into a Reproducing Kernel Hilbert Space (RKHS) [25]. Distances between subjects are then computed as distances between RKHS mean embeddings, under the principle of the Maximum Mean Discrepancy (MMD), yielding a tractable alternative to compare generative temporal models without reducing them to ad hoc concatenations of their parameters.

For a trained HMM with parameters $\theta_{s}=\left(\pi_{s},A_{s},\phi_{s}\right)$, let $\pi_{s}^{\mathrm{stat}}\in {\left[0,1\right]}^{K}$ denote its stationary state distribution that satisfies

(6) $${\left(\pi_{s}^{\mathrm{stat}}\right)}^{⊤}={\left(\pi_{s}^{\mathrm{stat}}\right)}^{⊤}A_{s},\;\sum_{i=1}^{K}\pi_{si}^{\mathrm{stat}}=1.$$

Under stationarity, the observation distribution induced by the HMM results from the marginalization over the latent states and becomes the following Gaussian mixture model with KM components:

(7) $$p\left(x\right)=\sum_{i=1}^{K}\pi_{si}^{\mathrm{stat}}\sum_{m=1}^{M}w_{sim}\;N\left(x|\mu_{sim},\Sigma_{sim}\right),$$

where the effective weight of component (i,m) is the product $\pi_{si}^{\mathrm{stat}}w_{sim}$. Thus, the HMM induces a stationary Gaussian mixture distribution in the observation space that summarizes the long-run subject-specific EEG dynamics while preserving the contribution of both the latent-state transition structure and the state-dependent emission densities.

The proposed methodology estimates pair-wise subject similarities based on their corresponding stationary distributions. To this end, the kernel mean embedding maps the subject’s stationary distribution $p_{s}\left(x\right)$ into a Reproduced-Kernel Hilbert Space (RKHS), where distances are computed between distributional representations. Given a positive-definite kernel $\kappa :R^{C^{*}}\times R^{C^{*}}\to R$ with associated RKHS H, the kernel mean embedding of $p_{s}\left(x\right)$ is defined as

(8) $$\mu_{s}=\int \kappa \left(x,\cdot \right)\;p_{s}\left(x\right)\;dx\in H.$$

Since κ(·,·) constitutes a characteristic kernel, the embedding in Equation (8) is injective and the distance between two embedded distributions in the RKHS defines a valid metric. Accordingly, the Hilbert embedding distance between two subjects *s* and $s^{\prime }$ is now given in terms of their mean respective mean embeddings:

(9) $$d_{H}\left(s,s^{\prime }\right)={∥\mu_{s}-\mu_{s^{\prime }}∥}_{H}.$$

As a distance, $d_{H}\left(\cdot ,\cdot \right)$ is non-negative, symmetric, satisfies the triangle inequality, and is zero only when the stationary observation distributions of corresponding subjects coincide. To obtain a computable expression for this distance, the squared RKHS distance is expanded in terms of inner products:

(10) $$d_{H}^{2}\left(s,s^{\prime }\right)={\langle \mu_{s},\mu_{s}\rangle }_{H}+{\langle \mu_{s^{\prime }},\mu_{s^{\prime }}\rangle }_{H}-2{\langle \mu_{s},\mu_{s^{\prime }}\rangle }_{H}.$$

According to the reproducing property, the cross-inner product between two embedded stationary distributions is

(11) $${\langle \mu_{s},\mu_{s^{\prime }}\rangle }_{H}=\;\int \int \;\kappa \left(x,x^{\prime }\right)p_{s}\left(x\right)p_{s^{\prime }}\left(x^{\prime }\right)\;dx\;dx^{\prime }.$$

Since $p_{s}$ and $p_{s^{\prime }}$ correspond to Gaussian mixture distributions, the integral in Equation (11) decomposes into weighted pairwise contributions between Gaussian components:

(12) $${\langle \mu_{s},\mu_{s^{\prime }}\rangle }_{H}=\sum_{i=1}^{K}\sum_{m=1}^{M}\sum_{i^{\prime }=1}^{K}\sum_{m^{\prime }=1}^{M}\pi_{si}^{\mathrm{stat}}\pi_{s^{\prime }i^{\prime }}^{\mathrm{stat}}w_{sim}w_{s^{\prime }i^{\prime }m^{\prime }}\;\hat{\kappa }\left(\mu_{sim},\mu_{s^{\prime }i^{\prime }m^{\prime }};\Sigma_{sim},\Sigma_{s^{\prime }i^{\prime }m^{\prime }},l\right),$$

with $\hat{\kappa }$ denoting the closed-form expectation of the base kernel between two Gaussian components:

(13) $$\hat{\kappa }\left(\mu ,\nu ;\Sigma ,\Lambda ,l\right)=\frac{|l^{2}I_{C^{*}}{|}^{1/2}}{|\Sigma +\Lambda \;+\;l^{2}I_{C^{*}}{|}^{1/2}}\exp\;\left(-\frac{1}{2}{\left(\mu -\nu \right)}^{\;⊤}{\left(\Sigma +\Lambda +l^{2}I_{C^{*}}\right)}^{-1}\left(\mu -\nu \right)\right),$$

l>0 standing for the kernel bandwidth, and $I_{C^{*}}$ as the $C^{*}\times C^{*}$ identity matrix. The expression in Equation (13) jointly accounts for the separation between component means and the uncertainty encoded by their covariance matrices. Consequently, the closer the means relative to their combined covariance structure and the RKHS bandwidth, the larger the similarity between their Gaussian components.

Hence, the pair-wise subject squared distance in Equation (10) can be computed as:

(14) $$\begin{array}{ccc} {\hat{d}}_{H}^{2}\left(s,s^{\prime }\right) & = & \sum_{i=1}^{K}\sum_{i^{\prime }=1}^{K}\sum_{m=1}^{M}\sum_{m^{\prime }=1}^{M}\pi_{si}^{\mathrm{stat}}\pi_{si^{\prime }}^{\mathrm{stat}}w_{sim}w_{si^{\prime }m^{\prime }}\hat{\kappa }\left(\mu_{sim},\mu_{si^{\prime }m^{\prime }};\Sigma_{sim},\Sigma_{si^{\prime }m^{\prime }}l\right)\\ \; & + & \sum_{i=1}^{K}\sum_{i^{\prime }=1}^{K}\sum_{m=1}^{M}\sum_{m^{\prime }=1}^{M}\pi_{s^{\prime }i}^{\mathrm{stat}}\pi_{s^{\prime }i^{\prime }}^{\mathrm{stat}}w_{s^{\prime }im}w_{s^{\prime }i^{\prime }m^{\prime }}\hat{\kappa }\left(\mu_{s^{\prime }im},\mu_{s^{\prime }i^{\prime }m^{\prime }};\Sigma_{s^{\prime }im},\Sigma_{s^{\prime }i^{\prime }m^{\prime }}l\right)\\ \; & - & 2\sum_{i=1}^{K}\sum_{i^{\prime }=1}^{K}\sum_{m=1}^{M}\sum_{m^{\prime }=1}^{M}\pi_{si}^{\mathrm{stat}}\pi_{s^{\prime }i^{\prime }}^{\mathrm{stat}}w_{sim}w_{s^{\prime }i^{\prime }m^{\prime }}\hat{\kappa }\left(\mu_{sim},\mu_{s^{\prime }i^{\prime }m^{\prime }};\Sigma_{sim},\Sigma_{s^{\prime }i^{\prime }m^{\prime }}l\right). \end{array}$$

The first two terms quantify the self-similarity of the stationary observation distributions associated with subjects *s* and $s^{\prime }$, whereas the third term measures their cross-similarity. For computational efficiency, the distance in Equation (14) is written compactly in matrix form [23]. To this end, let the stationary mixture-weight vectors $\alpha_{s}={\left[\pi_{si}^{\mathrm{stat}}w_{sim}\right]}_{i,m=1}^{K,M}$, where the indexing proceeds first over hidden states and then over Gaussian components within each state. The corresponding kernel matrices are defined as

(15) $$\begin{array}{cc} {\left[K_{ss}\right]}_{\left(im\right),\left(i^{\prime }m^{\prime }\right)}= & \hat{\kappa }\left(\mu_{sim},\mu_{si^{\prime }m^{\prime }};\Sigma_{sim},\Sigma_{s^{\prime }i^{\prime }m^{\prime }},l\right) \end{array}$$

(16) $$\begin{array}{cc} {}[K_{s^{\prime }s^{\prime }}{]}_{\left(im\right),\left(i^{\prime }m^{\prime }\right)}= & \hat{\kappa }\left(\mu_{s^{\prime }im},\mu_{s^{\prime }i^{\prime }m^{\prime }};\Sigma_{s^{\prime }im},\Sigma_{s^{\prime }i^{\prime }m^{\prime }},l\right) \end{array}$$

(17) $$\begin{array}{cc} {}[K_{ss^{\prime }}{]}_{\left(im\right),\left(i^{\prime }m^{\prime }\right)}= & \hat{\kappa }\left(\mu_{sim},\mu_{s^{\prime }i^{\prime }m^{\prime }};\Sigma_{sim},\Sigma_{s^{\prime }i^{\prime }m^{\prime }},l\right) \end{array}$$

with $K_{ss},K_{s^{\prime }s^{\prime }},K_{ss^{\prime }}\in R^{KM\times KM}$. The squared distance is then efficiently computed as

(18) $${\hat{d}}_{H}^{2}\left(s,s^{\prime }\right)=\alpha_{s}^{⊤}K_{ss}\alpha_{s}+\alpha_{s^{\prime }}^{⊤}K_{s^{\prime }s^{\prime }}\alpha_{s^{\prime }}-2\alpha_{s}^{⊤}K_{ss^{\prime }}\alpha_{s^{\prime }}.$$

Therefore, the matrix formulation constitutes the computational core of HIS, as it enables efficient construction of pairwise distances between all subject-specific HMM-induced stationary distributions.

### 2.6. Classification with Precomputed Kernels

After training an HMM–GMM model for each subject s∈S, HIS produces a pairwise distance matrix by comparing the RKHS embeddings of the corresponding HMM-induced stationary distributions according to Section 2.5. To test the discrimination capabilities of the proposed representation, the distances in Equation (18) feed two traditional similarity-based classifiers, namely, a *k*-Nearest Neighbors (KNN) and a Support Vector Machine (SVM), both operating in precomputed-kernel mode. Such a strategy enables classification directly in the geometric space induced by the generative models, without handcrafted feature engineering.

Since the square-root function is strictly monotonic over the non-negative real numbers, explicitly computing ${\hat{d}}_{H}\left(s,s’\right)$ is unnecessary for KNN and SVM classifiers. In the case of the KNN, the squared distances preserve exactly the same pairwise ordering while avoiding an additional square-root operation. Hence, the label assignment according to the majority vote among the *k* nearest neighbors is carried out on the precomputed distance matrix $D_{H}^{2}\in R^{\left|S|\times |S\right|}$ with elements ${\left[D_{H}^{2}\right]}_{ss^{\prime }}={\hat{d}}_{H}^{2}\left(s,s^{\prime }\right)$, |S| as the number of subjects in the dataset, and *k* controlling the neighborhood size and the decision boundary complexity. In the case of SVM, since the traditional gaussian kernel is parameterized by a squared distance, the required positive semi-definite Gram matrix is computed from Equation (18) with elements ${\left[K_{H}\right]}_{ss^{\prime }}=\exp\left(-{\hat{d}}_{H}^{2}\left(s,s^{\prime }\right)/2l\right)$, where the kernel bandwidth parameter l controls the similarity decay rate. Then, the maximum-margin classifier is trained using the dual formulation in Equation (19) with the precomputed kernel matrix $K_{H}$:

(19) $$\begin{array}{cc} \max_{\alpha \in R^{N_{\mathrm{tr}}}}\; & \sum_{s\in S_{\mathrm{tr}}}\alpha_{s}-\frac{1}{2}\sum_{s\in S_{\mathrm{tr}}}\sum_{s^{\prime }\in S_{\mathrm{tr}}}\alpha_{s}\alpha_{s^{\prime }}y_{s}y_{s^{\prime }}{\left[K_{H}\right]}_{ss^{\prime }}\\ \mathrm{subject}\;\mathrm{to}\; & 0\leq \alpha_{s}\leq C,\;\forall s\in S_{\mathrm{tr}},\\ \; & \sum_{s\in S_{\mathrm{tr}}}\alpha_{s}y_{s}=0, \end{array}$$

with $\alpha_{s}$ as the dual coefficient associated with subject *s* and C>0 controlling the trade-off between margin maximization and training errors. Then, for an unseen subject *r*, the posterior probability for ADHD class is computed following the Platt scaling:

(20) $$p\left(y_{r}=1|r\right)=\frac{1}{1+\exp\;\left(af(r)+b\right)},$$

being *a* and *b* the sigmoid parameters fit by an additional cross-validation on the training data, and f(r) the signed decision function:

(21) $$f\left(r\right)=\sum_{s\in S_{\mathrm{tr}}}\alpha_{s}^{★}y_{s}{\left[K_{H}\right]}_{sr}+b^{★}$$

where only subjects satisfying $\alpha_{s}^{★}>0$ act as support vectors and $b^{★}$ is the optimal intercept. During validation and testing, the precomputed kernel contains similarities between each held-out subject and the training subjects; therefore, no similarities among held-out subjects are used to fit the classifier. Consequently, the predicted class is then obtained according to

(22) $${\hat{y}}_{r}=\left\{\begin{array}{cc} 1, & p(y_{r}=1|r)\geq 0.5,\\ -1, & \mathrm{otherwise}. \end{array}\right.$$

## 3. Results

To assess the proposed HMM-Induced Stationary (HIS) RKHS distance, we benchmark it against the Probability Product Kernel (PPK) between HMMs [36], a previously validated probabilistic kernel for comparing generative EEG models in ADHD diagnosis [24]. Whereas HIS embeds each HMM through its stationary observation distribution, PPK approximates similarity over a finite horizon by truncating state-transition propagation at *T*. For a fair comparison, both kernels used the same subject-level partitions, cross-validation protocol, and learned subject-wise HMMs, with hyperparameters selected within the training folds. The comparison thus tests whether the stationary-distribution formulation represents long-run HMM behavior more reliably than a finite-horizon similarity.

### 3.1. Performance Analysis on the Synthetic Dataset

Since the synthetic dataset is balanced with 50 recordings per class, accuracy becomes the primary metric, complemented by balanced accuracy, AUC-ROC, and the Matthews correlation coefficient (MCC). HMMs are trained with K=3 states and M=3 Gaussian components. The dataset $D^{\mathrm{synth}}$ is then partitioned at the recording level into training and test subsets, comprising 80% and 20% of the data, respectively. Stratified random sampling preserves class proportions across both subsets, and the training subset is further divided into seven stratified folds for cross-validation. Hyperparameters for both classifiers are optimized using Bayesian optimization with the Optuna framework [37], using mean validation accuracy across the seven folds as the objective function. The search spaces are $l\in [10^{-2},10^{0}]$ for the RKHS kernel bandwidth, $C\in [10^{-2},10^{2}]$ for the SVM box-constraint regularization parameter, and k∈{1,3,5,7,9,11} for the number of neighbors, restricted to odd values given the binary classification task. Once Bayesian optimization identifies the best hyperparameters, the classifiers are retrained on the full training subset and evaluated once on the held-out test subset. This protocol confirms that the proposed approach and classifiers recover the artificial, pre-specified class structure built into the data-generation process. As reported in Table 1, HIS outperforms PPK across all classifiers, subsets, and metrics, with SVM-HIS achieving the best result (95.0% test accuracy and balanced accuracy). In particular, HIS improves test and balanced accuracy by 5 percentage points for both classifiers, confirming that the stationary kernel captures class-discriminative information more effectively than PPK under controlled conditions.

**Figure 4:**
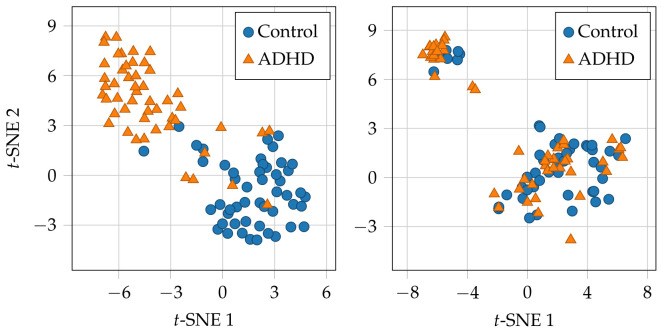
t-SNE projection of the proposed HIS (**left**) and the baseline PPK (**right**) kernel matrices computed on the synthetic EEG dataset and HMM–GMM models with three states and three Gaussian components.

For visual interpretation, Figure 4 presents t-SNE projections of the pairwise RKHS-based kernel matrices obtained with the proposed HIS similarity (left) and the baseline PPK similarity (right) on the synthetic EEG dataset. The HIS embedding exhibits a clear separation between the two classes even after projection onto a two-dimensional manifold. ADHD subjects form a compact cluster predominantly in the upper-left region, whereas Control subjects are concentrated in the lower-right region, with only a small overlap near the origin. This overlap is consistent with the few misclassified samples observed during cross-validation and suggests that most subjects are already well separated in the RKHS induced by the proposed stationary-distribution representation before classifier training. In contrast, the PPK embedding displays noticeably stronger class mixing and a less structured geometry, indicating that its finite-horizon similarity captures the underlying HMM differences less effectively. Although t-SNE is intended solely as a qualitative visualization and does not preserve global distances, the markedly improved separation obtained with HIS is consistent with its superior quantitative performance reported in Table 1. Hence, the intuitive geometric embedding evidences that the proposed distance reveals the class-discriminative structure of the HMM parameter space more effectively than the finite-horizon PPK formulation.

### 3.2. Hyperparameter Tuning

On the real EEG, three HMM sizes were evaluated (K,M∈{3,4,5}). To evidence HMM convergence, Table 2 reports the percentage of subjects whose BW fit terminated under each criterion, by group and model size. Across all six group/size combinations, stationary-distribution convergence is the dominant stopping criterion (72–97% of subjects), with validation-segment log-likelihood convergence accounting for the remainder (2–21%). Algorithm stopping due to the maximum-iteration budget without satisfying either convergence criterion is rare (0–2% of subjects), occurring at most once per group/configuration, and outright numerical errors did not occur in any configuration. Aggregated across all three topologies, 115/121 subjects (95%) converged via one of the two genuine criteria, consistent with the per-configuration breakdown below. Log-likelihood convergence is comparatively more frequent under K = M = 3 in both groups (Control 12%, ADHD 21%) than under K = M = 4 or K = M = 5, consistent with a smaller parameter space reaching a log-likelihood plateau before the stationary distribution has fully settled. No consistent group-level asymmetry in convergence behavior is observed: the ADHD group shows a wider split between criteria under K = M = 3 but converges predominantly via the stationary-distribution criterion under K = M = 4 and K = M = 5, mirroring the Control group. This indicates that differences in HMM-fitting quality are unlikely to confound the downstream ADHD vs. Control separation.

Given that the hyperparameter search must be repeated independently for each of the 25 outer folds, across both kernel families, classifiers, and HMM sizes, an exhaustive grid search ensures deterministic and reproducible model selection. For the SVM classifier, two bandwidth parameters are distinguished explicitly: the HIS bandwidth $l\in \{10^{-3},10^{-2.5},10^{-2},10^{-1.5},10^{-1},10^{-0.5},10^{0}\}$, shared with KNN and used to construct the underlying HIS embedding and distance, and the RBF kernel bandwidth $\gamma \in \{10^{-4},10^{-3.4},10^{-2.8},10^{-2.2},10^{-1.6},10^{-1}\}$, which is applied only during the exponential kernel transformation used by the SVM classifier. Regarding the classifiers-dependent hyperparameters, the number of neighbors for the KNN is explored within k∈{1,3,5,7,9}, while the regularization parameter for the SVM is tuned within the grid $C\in \{10^{-2},10^{-1},10^{0},10^{1},10^{2},10^{3},10^{4}\}$. This search yields 35 candidate hyperparameter configurations for KNN and 210 for SVM, independently evaluated within each outer training partition. Final performance is aggregated over the 25 outer test folds and reported using bootstrap 95% confidence intervals for accuracy, balanced accuracy, sensitivity, specificity, AUC-ROC, and Matthews correlation coefficient (MCC), together with pooled confusion matrices and a label-permutation significance test (n=1000 permutations). Consequently, the tuning protocol attempts to estimate the true predictive performance expected on previously unseen subjects, accounting for uncertainty in hyperparameter selection, thereby providing evidence for the generalization capability of the proposed methodology.

**Figure 5:**
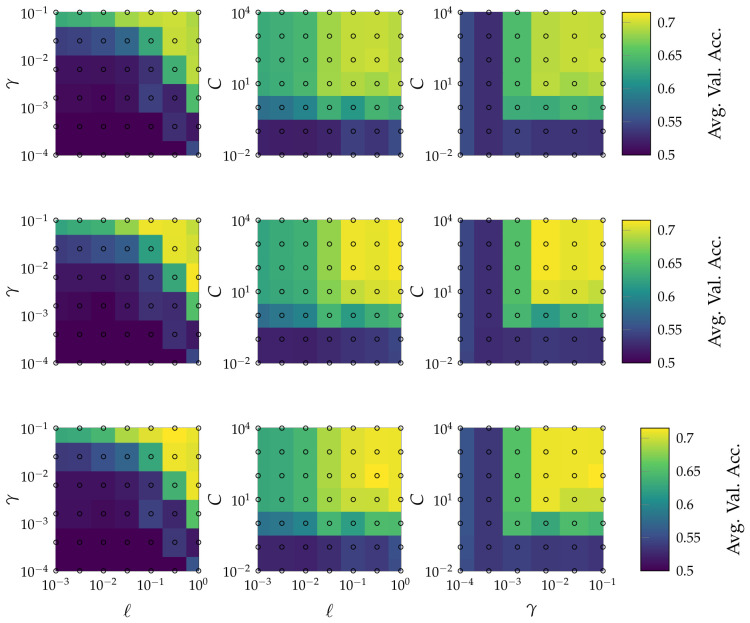
Hyperparameter performance landscapes for the HIS distance within SVM classifier. Top to bottom: HMM sizes of K=M=3, K=M=4, and K=M=5. Mean validation accuracy for nested cross-validation is color-coded.

Particularly for the SVM classifier, Figure 5 illustrates the hyperparameter performance landscapes under the nested cross-validation protocol, displayed as three two-dimensional projections of the three-dimensional search space across the HIS kernel bandwidth l, the SVM RBF envelope γ, and the box-constraint regularization *C*, for the three considered HMM sizes (top to bottom: 3, 4, 5). Each cell color-codes the mean validation accuracy averaged over the inner cross-validation folds, ranging from chance level (dark purple) to the maximum observed (yellow). Beyond only identifying the single best hyperparameter configuration, these landscapes characterize the topology of the optimization surface and reveal whether the classifier performance is sensitive to small hyperparameter perturbations or remains stable across extended regions of the search space.

Firstly, the landscapes exhibit a structured optimization surface with clearly identifiable high-performance plateaus. Across all HMM sizes (3, 4, and 5), the regularization parameter *C* and the RBF kernel parameter γ dominate the classifier behavior. Low values of γ consistently produce poor balanced accuracy regardless of the remaining parameters, indicating that an excessively smooth decision boundary is unable to capture the nonlinear class structure embedded in the HIS representation. As γ increases toward intermediate values, performance rapidly improves and subsequently reaches a relatively broad plateau, particularly when combined with moderate-to-large values of *C*. This behavior suggests that once the kernel is sufficiently localized, the classifier becomes considerably less sensitive to further adjustments of the regularization parameter. The resulting plateau is desirable because it indicates that the optimal operating region is not confined to a single isolated configuration but instead spans a relatively wide portion of the hyperparameter space.

Secondly, the interaction between l and γ illustrates the complementary roles of the two parameters. The parameter l, which governs the scale of the HIS distance kernel used to construct the similarity representation, influences the separability of the embedded samples prior to classification. The landscapes show that small values of l generally yield lower performance unless compensated by sufficiently large values of γ. Conversely, intermediate values of l produce consistently higher balanced accuracy over a broad range of kernel parameters, suggesting that the proposed HIS representation becomes stable once an appropriate similarity scale has been selected. This observation indicates that the HIS embedding contributes substantially to the discriminative structure, while the SVM primarily refines an already informative representation.

Thirdly, the (l,C) landscapes evidence that performance changes smoothly along the first one, whereas the transition from small to moderate values of the second one produces a much stronger increase in balanced accuracy. After this transition, additional increases in *C* provide only marginal improvement, indicating diminishing returns from increasingly strict margin optimization. Such behavior is characteristic of classifiers operating on well-separated feature representations, where the margin is already sufficiently discriminative and further regularization is unnecessary.

A last important observation is the consistency of the landscapes across HMM sizes. Although the absolute balanced accuracy increases slightly with the number of states, the overall geometry of the optimization surface remains remarkably similar. The location of the high-performance regions changes only marginally, indicating that the HMM size primarily shifts the achievable performance level rather than altering the optimal hyperparameter regime. This stability suggests that the proposed HIS representation produces consistent similarity structures regardless of the number of states, thereby reducing the need for extensive hyperparameter tuning.

### 3.3. Performance Analysis on the Real EEG Dataset

For a quantitative analysis, Table 3 summarizes classification performance under the nested cross-validation framework (5 outer folds × 5 repeats) for every combination of HMM size, kernel function, and classifier. The table compares the proposed HIS kernel with the baseline PPK kernel, both combined with KNN and SVM classifiers. Table 3 also compares the proposed approach against the work of Alim and Imtiaz [32] as a controlled feature-based baseline. The baseline was reimplemented in-house and trained under the same experimental conditions used throughout this work, including the same seven frontal EEG channels, subject-level grouped nested cross-validation, identical train/test partitioning strategy, and subject-level decision aggregation. The original feature representation was preserved, comprising the same 11 statistical, Hjorth, and spectral descriptors computed over the four canonical sub-bands (delta, theta, alpha, and beta). The complete source code of the reimplementation has been included in the public repository accompanying this article to facilitate verification and reproducibility. For each contrasted approach, mean performance metrics are reported across the 25 outer folds of the test set. Balanced accuracy, sensitivity, specificity, AUC-ROC, and Matthews correlation coefficient (MCC) are reported, with 95% bootstrap confidence intervals for balanced accuracy and MCC computed from 2000 resamples. The statistical significance of each configuration is assessed using a label-permutation test with 1000 permutations. These metrics provide an unbiased estimate of out-of-sample performance after accounting for both hyperparameter-selection uncertainty and sampling variability.

Results from Table 3 confirm the optimal configuration of the original feature-based baseline, with 90% retained variance PCA for dimensionality reduction and an RBF-SVM for classification. Moreover, the label-permutation test indicates that the observed discrimination is significantly above chance, thereby verifying the discriminative capacity of the features. Nevertheless, the overall accuracy decreases from approximately 93% in the original work to 68% in the reproduced version, attributable to two methodological differences. First, the considered validation mitigates the information leakage by grouping all segments from the same subject within a single fold. Second, the subject-balanced evaluation prevents longer ADHD recordings from dominating the optimization process. In turn, all evaluated HMM-based configurations achieve statistically significant performance under the label-permutation test, demonstrating that both kernel formulations extract discriminative information beyond chance. The proposed HIS kernel combined with SVM provides the strongest overall performance, achieving the best balanced accuracy, AUC-ROC, and MCC for a compact HMM topology (K=M=3). Increasing the HMM complexity from three to five hidden states produces only marginal changes in predictive performance, with the best balanced accuracy remaining within a narrow range of 73.0–73.5%. Although the relative performance of HIS and PPK depends on the classifier, the best-performing model results from the proposed stationary-distribution kernel, suggesting that its representation captures the long-term probabilistic structure of subject-specific HMMs more effectively than the finite-horizon PPK formulation. Consequently, the HIS–SVM configuration with K=M=3 is adopted as the reference model for the remainder of this work, as it provides the best balance among predictive performance, statistical robustness, and topology simplicity in the nested cross-validation framework.

**Figure 6:**
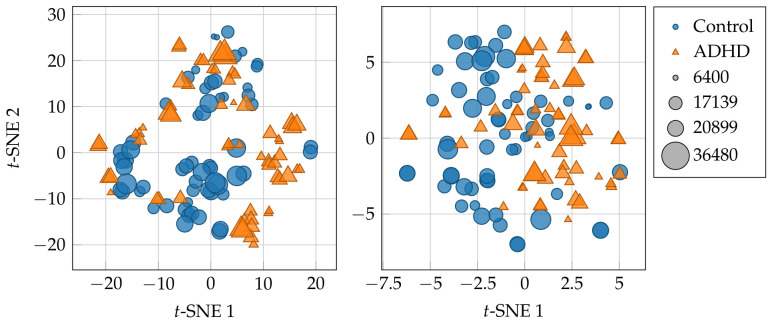
Two-dimensional t-SNE mapping of subjects based on the pairwise kernel matrices for the proposed HIS (**left**) and the baseline PPK (**right**) kernel. The kernel is computed for the K=M=3 HMM configuration. Perplexity is set to 10 (HIS) and 50 (PPK). Marker size encodes the length of the EEG recording in terms of the number of samples.

To complement the quantitative results, Figure 6 shows t-SNE projections of the HIS and PPK kernel matrices on the real data using visually tuned perplexities for each kernel. Each marker denotes a subject with shape and color representing the class. Marker size is proportional to the length of the EEG recording, as described in the legend, in terms of the number of samples. Because t-SNE preserves local neighborhoods, nearby subjects are locally similar under the corresponding kernel. Mainly, the HIS projection is broader and more structured, with several class-consistent local neighborhoods despite incomplete global separation, whereas PPK is more compact and diffuse, with classes more frequently intermixed, suggesting more homogeneous pairwise similarities that shift the discriminative burden to the classifier. This matches the formulation of each kernel, namely, stationary distributions vs. finite-horizon truncation. The broader dispersion among ADHD subjects, including more variable recording durations, is plausible given the heterogeneity of attentional engagement in pediatric ADHD [38]. Therefore, the improved performance of HIS stems from a more informative kernel-induced representation, in which ADHD-related similarities are encoded before any supervised classifier is applied.

Since Figure 2 illustrates longer recordings for children with ADHD, a potential confounding factor may affect the performance of the proposed HIS. An ordinary least-squares (OLS) linear regression analysis was performed to quantify the proportion of variance in prediction confidence explained by recording duration. Prediction confidence is defined for the *s*-th subject as $p_{\mathrm{CONF}}\left(s\right)=2\left|\bar{p}\left(y_{s}=1|s\right)-0.5\right|$, where ${\bar{p}}_{s}\left(y_{s}=1|s\right)$ denotes the subject-level averaged posterior probability for ADHD ($y_{s}=1$) resulting from Equation (20). $p_{\mathrm{CONF}}=0\%$ and $p_{\mathrm{CONF}}=100\%$ indicate complete uncertainty and maximum prediction certainty, respectively. Because the interpretation of the posterior probability differs between ADHD and Control subjects, the analysis was performed separately for each class.

**Figure 7:**
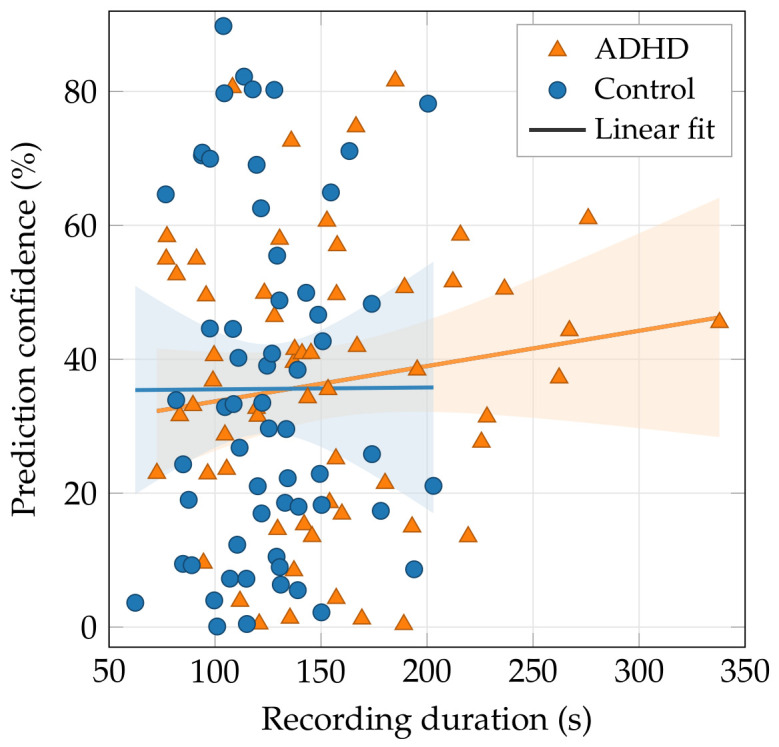
Class-wise ordinary least-squares regression analysis of recording duration versus subject-level prediction confidence. Solid lines denote the fitted linear regression models for the ADHD and Control groups, while the shaded regions correspond to the 95% confidence intervals of the estimated mean regression functions.

Figure 7 presents the OLS regression results for each class, with solid lines denoting the fitted linear regression models for the ADHD and Control groups matching colors with the corresponding class. The shaded regions correspond to the 95% confidence intervals of the estimated mean regression functions. Qualitatively, the scatter distributions exhibit no discernible linear trend in either diagnostic group, with the regression lines remaining nearly horizontal across the observed range of recording durations. Although ADHD subjects show a slightly positive slope, the corresponding regression model explained only 2.1% of the variance in prediction confidence, with the slope lacking statistical significance (p=2.61%). For the Control group, the fitted model indicated that classifier confidence remained consistent regardless of recording duration ($R^{2}<0.001$, p=98%). The wide overlap of confidence values across short and long recordings, together with the broad confidence intervals of the fitted regression functions, further indicates that trends lie within the uncertainty of the estimated models. Therefore, the recording duration explains only a negligible proportion of the variance in the subject-level posterior confidence, concluding that the classifier confidence is primarily driven by the HMM-derived representation rather than by differences in EEG recording length.

To contextualize the work within the state of the art, Table 4 compares recent EEG-based ADHD classification methods evaluated on the same public benchmark dataset. Beyond the reported accuracy, the comparison includes the learning algorithm, data representation, classification unit, and validation protocol, all of which substantially influence the reliability and interpretation of the reported performance. It is worth noting that although reported accuracies range from 73.5% to 100.0%, these values should be carefully compared because the studies differ considerably in cohort size, sample generation strategies, and evaluation methodologies.

Several studies report remarkably high accuracies using segment-level classification. Khare and Acharya [15], Loh et al. [14], Han et al. [40], and Ahire [21] classify short EEG segments using handcrafted features or deep neural networks, achieving accuracies above 97%. However, segment-based evaluation substantially increases the number of available training samples and, unless subject independence is rigorously enforced, may allow multiple segments from the same child to appear in both the training and validation partitions. Consequently, these protocols primarily assess the ability to recognize previously observed recording characteristics rather than to generalize to unseen subjects. Nouri and Tabanfar [39] partially addressed this limitation through subject-wise majority voting over 3-s segments, although the model was evaluated on only 61 children.

Subject-level classification provides a more clinically meaningful evaluation because each prediction corresponds to an individual child rather than to isolated EEG windows. Chauhan and Choi [18], Maniruzzaman et al. [17], García-Ponsoda et al. [20], and Fayyazi et al. [11] perform subject-level prediction using regional channel representations, handcrafted statistical features, Gaussian processes, or functional-connectivity descriptors. Nevertheless, their validation strategies vary substantially. Chauhan and Choi [18] lack a subject-partition declaration; Maniruzzaman et al. [17] employed a single 20% holdout split; García-Ponsoda et al. [20] adopted conventional 5-fold cross-validation; and Fayyazi et al. [11] reported perfect accuracy using repeated 10-fold cross-validation on only 116 subjects. These methodological differences make direct numerical comparisons difficult because the reported accuracy depends not only on the classifier but also on the strictness of the evaluation protocol.

The proposed HIS framework differs from previous approaches in both representation learning and experimental validation. Instead of extracting handcrafted EEG descriptors or generating thousands of short temporal segments, HIS models each child using a single subject-specific HMM–GMM estimated from the complete EEG recording. Classification is therefore performed directly at the subject level using probabilistic representations of temporal brain dynamics, naturally accommodating recordings of variable duration without sample augmentation. More importantly, model selection is performed within an inner cross-validation loop, while final performance is estimated exclusively on unseen outer folds using repeated nested 5 × 5 cross-validation, yielding 25 independent outer-fold evaluations. Among the studies summarized in Table 4, this work presents the most rigorous evaluation protocol because it simultaneously prevents information leakage during hyperparameter optimization and reduces the dependence of the reported accuracy on a particular train-test partition. Although the resulting balanced accuracy is lower than previously reported values, the HIS performance is interpreted in the context of a demanding validation procedure. Unlike point estimates obtained from a single experimental partition, the repeated nested protocol provides an approximately unbiased estimate of the expected generalization performance together with an associated confidence interval, thereby quantifying the variability introduced by different subject partitions. Consequently, the contribution of the proposed HIS framework extends beyond predictive accuracy by establishing a reproducible, leakage-aware benchmark that combines probabilistic modeling, subject-level inference, and statistically robust evaluation. Hence, the nested and repeated subject-independent validation protocol provides conservative performance estimates that offer a realistic assessment of the clinical potential of EEG-based ADHD identification systems.

## 4. Discussion

This work proposed an HMM-Induced Stationary (HIS) RKHS distance for subject-level EEG-based ADHD classification in which each child is represented by a subject-specific HMM–GMM and compared through a novel RKHS-based distance derived from stationary observation distributions. The proposed similarity measure was theoretically formulated as a valid metric between HMMs, efficiently implemented via a kernel matrix representation, and evaluated on synthetic and real EEG datasets against the Probability Product Kernel (PPK) under a rigorous validation protocol that included repeated nested cross-validation. Under this demanding evaluation protocol, the proposed HIS–SVM model with K=M=3 achieved a balanced accuracy of 73.5% (95% CI [69.8,77.0], MCC =0.483, permutation p<0.001), computed from 605 independent subject-level predictions across 25 outer folds. This estimate constitutes the primary measure of the expected generalization performance of the proposed framework. Although lower than many published accuracies summarized in Table 4, this result is interpreted in light of a substantially strict evaluation methodology. Most previous studies rely on segment-level classification, single holdout partitions, or conventional cross-validation without nested hyperparameter optimization, all of which tend to produce more optimistic estimates. By separating hyperparameter tuning from testing and averaging performance across repeated subject-independent partitions, the nested cross-validation protocol minimizes information leakage and provides a statistically robust estimate of real-world generalization.

Beyond predictive performance, the experiments support the theoretical motivation underlying the proposed similarity measure. HIS compares HMMs through RKHS embeddings of their stationary observation distributions, whereas PPK compares finite-horizon transition probabilities. Consequently, HIS characterizes the long-run probabilistic behavior of each subject without requiring an arbitrary truncation horizon, emphasizing persistent temporal EEG dynamics rather than transient state trajectories. This theoretical advantage is consistently reflected across the experiments, in which HIS outperforms PPK on both synthetic and real datasets under every evaluation protocol considered. Furthermore, the repeated selection of the most compact topology (K=M=3) indicates that a small number of latent states adequately captures the dominant temporal organization of frontal EEG, whereas more complex HMMs increase estimator variance without introducing additional discriminative information. Collectively, these observations suggest that the representation itself, rather than classifier complexity, constitutes the primary source of the observed performance gains.

The complementary analyses further explain this behavior. The hyperparameter landscapes (Figure 5) reveal broad high-performance regions for the proposed HIS representation at K=M=3, indicating that the induced RKHS similarities remain stable over a wide range of kernel parameters. As HMM complexity increases, these regions progressively fragment, suggesting that larger models begin to capture subject-specific variability rather than population-level ADHD characteristics. Likewise, the t-SNE visualization (Figure 6) shows that HIS preserves more coherent local neighborhoods than PPK, despite the absence of complete global class separation. This behavior is consistent with the local margin optimization performed by SVMs and provides a qualitative explanation for the improved classification performance. Moreover, the class-wise OLS analysis found that duration explained virtually none of the variation in prediction confidence. This result suggests that confidence was not strongly determined by sequence length within either diagnostic group, indicating that the proposed similarity primarily reflects temporal EEG dynamics rather than recording duration.

From a neurophysiological perspective, the results indicate that a restricted frontal montage contains temporal information to distinguish children with ADHD from typically developing controls. The selected electrodes are closely associated with executive function and attentional regulation, domains consistently impaired in ADHD [33]. Unlike conventional feature-based approaches that quantify isolated spectral or statistical characteristics, the HMM representation models transitions between latent neural regimes, implicitly capturing the temporal organization of frontal brain activity throughout the recording. The broader dispersion observed among ADHD subjects in the induced kernel space is consistent with the well-documented heterogeneity of attentional engagement and increased neural variability associated with the disorder [29,38]. Although the current quality-control procedure removes recordings with anomalous amplitude and variance, future studies incorporating dedicated ocular-artifact correction and multimodal physiological recordings may further refine the estimated subject representations.

## 5. Concluding Remarks and Future Directions

This study introduced HIS, an HMM-Induced Stationary RKHS framework for discriminating between ADHD and Control EEG recordings from frontal-channel recordings. The proposed methodology represents each participant with a subject-specific HMM–GMM and compares them by using the RKHS squared distance between the stationary observation distributions induced by their corresponding probabilistic models. By operating directly on subject-level generative representations, HIS avoids handcrafted feature engineering while preserving the temporal structure learned from the EEG recordings. The resulting framework combines probabilistic modeling, kernel-based similarity learning, and subject-level classification within a unified pipeline intended to support clinical assessment.

The experimental evaluation demonstrated that the proposed stationary-distribution representation provides a statistically robust alternative to existing HMM similarity measures. On the synthetic benchmark, HIS consistently outperformed the Probability Product Kernel by five percentage points, confirming that the proposed distance effectively captures discriminative temporal structure under controlled conditions. On the real pediatric EEG dataset, rigorously evaluated via nested cross-validation, the compact configuration with K=M=3 achieved a balanced accuracy of 73.5% and an MCC of 0.483.

Beyond its predictive performance, the proposed framework offers several methodological advantages. Subject-level inference naturally avoids the information leakage frequently associated with segment-wise validation strategies while accommodating EEG recordings of unequal duration without requiring temporal segmentation or sample augmentation. The complementary analyses indicate that the learned kernel representation is primarily driven by the probabilistic characterization of EEG dynamics rather than by recording duration, and the t-SNE visualizations reveal a more structured and class-consistent representation than that obtained with the finite-horizon PPK formulation.

Taking into account the achieved results and the evidenced limitations, some opportunities exist to further strengthen the proposed methodology. Future work should investigate alternative electrode montages, including the complete 19-channel configuration, and evaluate additional baseline methods under identical preprocessing pipelines, subject-level partitions, and nested cross-validation protocols to guarantee fair comparisons. The latent HMM parameters, including transition probabilities, stationary-state occupancies, and Gaussian emission statistics, should be systematically analyzed to assess their neurophysiological relevance and potential as interpretable biomarkers. Furthermore, validation in larger multicenter cohorts, particularly those including medication-naive participants, and on external test datasets will be essential to establish the generalizability of the proposed framework. Finally, integrating more comprehensive artifact-removal procedures, adaptive, subject-specific model selection, multiple EEG acquisition paradigms, and more realistic synthetic benchmarks that incorporate physiological artifacts and spatial brain models constitutes a promising direction for extending the robustness and clinical applicability of the proposed HIS framework.

## Figures and Tables

**Table 1:** Classification performance on the synthetic EEG dataset using two similarity-based classifiers and the contrasted kernel-based similarities. CV metrics are reported as mean ± standard deviation over seven cross-validation folds. Test metrics correspond to the held-out test set. MCC is reported as a unitless coefficient in [−100,100].

Classifier	Kernel	Cross-Validation				Test Set			
		Acc (%)	BalAcc (%)	AUC (%)	MCC	Acc (%)	BalAcc (%)	AUC (%)	MCC
KNN	PPK	86.3±4.3	86.0±4.6	86.0±11.5	74.6±8.1	90.0	90.0	92.5	80.0
	HIS	89.9±8.8	90.0±8.7	92.0±6.9	80.6±17.4	95.0	95.0	100.0	90.5
SVM	PPK	88.8±6.0	88.6±6.3	93.5±6.1	79.9±10.6	90.0	90.0	97.0	80.0
	HIS	92.4±6.1	92.4±6.2	94.0±6.2	85.5±12.5	95.0	95.0	99.5	90.5

**Table 2:** Percentage of subjects according the BW-algorithm stopping criteria by group and HMM size.

Group	HMM Size	Convergence Criterion		
		Log-Likelihood	Stationary π	Max. Iterations
Control (n=60)	K=M=3	8 (13.3%)	51 (85.0%)	1 (1.7%)
	K=M=4	3 ( 5.0%)	56 (93.3%)	1 (1.7%)
	K=M=5	6 (10.0%)	54 (90.0%)	0 (0.0%)
ADHD (n=61)	K=M=3	13 (21.3%)	46 (75.4%)	2 (3.3%)
	K=M=4	2 ( 3.3%)	57 (93.4%)	2 (3.3%)
	K=M=5	1 ( 1.6%)	60 (98.4%)	0 (0.0%)

**Table 3:** Nested cross-validation performance comparison: HMM-based kernels versus baseline classifiers. Means over five outer folds and five repeats are presented. Balanced accuracy and MCC are reported with bootstrap 95% confidence intervals (n=2000 resamples). The rightmost column reports the label-permutation significance test (n=1000 permutations). Full per-configuration tables, including fold-level minimum/maximum and pooled confusion matrices, are provided in the project’s public code repository.

Model	Classifier	Kernel	Acc	BalAcc [95% CI]	Sens	Spec	AUC	MCC [95% CI]	Perm. *p*
Baseline [32]	KNN	—	59.7	59.4[55.5,63.1]	83.2	35.7	66.0	22.5[13.8,30.6]	0.01
	SVM	RBF	68.3	68.2[64.4,72.1]	78.1	58.3	73.9	38.2[30.3,46.1]	<0.001
HMM K=M=3	KNN	PPK	72.3	72.3[68.2,76.3]	79.9	64.7	76.5	45.6[37.2,53.7]	<0.001
		HIS	66.4	66.3[63.0,69.8]	70.7	62.0	73.1	33.9[26.8,41.1]	<0.001
	SVM	PPK	70.6	70.6[66.6,74.6]	76.8	64.3	76.2	42.5[34.5,50.8]	<0.001
		HIS	73.5	73.5[69.8,77.0]	77.3	69.7	79.6	48.3[40.6,55.2]	<0.001
HMM K=M=4	KNN	PPK	70.7	70.6[67.4,74.0]	75.9	65.3	76.6	42.8[36.4,49.6]	<0.001
		HIS	68.6	68.5[64.3,72.4]	75.3	61.7	76.4	38.6[30.2,46.7]	<0.001
	SVM	PPK	71.4	71.4[68.0,74.9]	78.0	64.7	76.7	44.2[37.7,51.2]	<0.001
		HIS	72.5	72.5[68.0,76.5]	77.4	67.7	81.3	45.8[36.6,53.9]	<0.001
HMM K=M=5	KNN	PPK	71.7	71.6[69.1,74.3]	76.6	66.7	77.8	45.0[39.7,50.3]	<0.001
		HIS	70.6	70.5[67.2,73.9]	76.3	64.7	74.9	42.1[35.2,49.0]	<0.001
	SVM	PPK	73.0	73.0[69.0,77.0]	77.7	68.3	80.0	47.1[39.2,55.3]	<0.001
		HIS	71.2	71.2[66.8,75.3]	72.7	69.7	79.9	43.1[34.3,51.6]	<0.001

**Table 4:** State-of-the-art studies using the same public EEG dataset. CV: cross-validation. DT: decision tree. NN: neural network. Accuracy values are reported as by their respective authors. The proposed HIS performance is accuracy with a bootstrap 95% confidence interval.

Study	Year	Classifier	Data Representation	Classification Unit	Validation Scheme	Accuracy (%)
Alim and Imtiaz [32]	2023	Gaussian SVM	Linear features from canonical sub-bands	2-s segment	10% holdout on 120 subjects	93.2
Chauhan and Choi [18]	2023	Naive Bayes	Regional channel-group representation	Subject	Unspecified data partitions on 120 subjects	84.0
Maniruzzaman et al. [17]	2023	Gaussian Process	Time-domain, morphological, and nonlinear features	Subject	20% holdout on 121 subjects	97.5
Khare and Acharya [15]	2023	Explainable Boosting Machine	Analytic mode functions	4-s segment	10-fold CV on 121 subjects	99.8
Nouri and Tabanfar [39]	2024	Convolutional NN	Spectral channel-frequency	Subject-wise majority voting from 3-s segments	5-fold CV on 61 subjects	94.5
Loh et al. [14]	2024	Convolutional NN	Channel-wise CWT correlation matrices	4-s segment	10-fold CV on 121 subjects	98.2
Han et al. [40]	2024	LSTM+Attention	Nonlinear, spectral, and channel-correlation	Segments of unspecified length	10-fold CV on 61 subjects	98.9
García-Ponsoda et al. [20]	2024	SVM, KNN, XGBoost	Statistical, nonlinear, and spectral features	Subject	5-fold CV on 121 subjects	86.1
Fayyazi et al. [11]	2025	DT, NN, and ensemble models	Functional connectivity	Subject	Repeated 10-fold CV on 116 subjects	100.0
Ahire [21]	2025	Attention Convolutional NN	Time-domain, frequency-domain, and statistical features	Segments of unspecified length	20% holdout on 121 subjects	97.6
Salazar-Dubois et al. [41]	2025	Attention NN	Gaussian kernel connectivity	4-s segment	Subject-wise 5-fold CV on 121 subjects	82.1
Pastrana-Cortés et al. [42]	2026	Attention pooling	Data-driven spatiotemporal convolutional embeddings	Subject-wise majority voting from 2-s segments	5-fold nested stratified group cross-validation	87.5
**Proposed HIS**	**2026**	**SVM**	**Subject-specific HMM–GMMs**	**Subject**	**Nested CV (5×5, 25 outer folds)**	**73.5 [69.8, 77.0]**

## Data Availability

The dataset EEG data for ADHD / Control children, used in the validation of the current study, is publicly available at https://ieee-dataport.org/open-access/eeg-data-adhd-control-children. The code, baseline reimplementation, full per-configuration results, and trained model artifacts supporting the findings of this study are openly available in the project’s repository (https://github.com/leonlpz/HMM_RKHS_Classification_Project (accessed on 19 May 2026)).
